# Vertebral body and splenic irradiation are associated with lymphopenia in localized pancreatic cancer treated with stereotactic body radiation therapy

**DOI:** 10.1186/s13014-021-01969-1

**Published:** 2021-12-24

**Authors:** Abhinav V. Reddy, Matthew P. Deek, Juan F. Jackson, Colin S. Hill, Shuchi Sehgal, Jin He, Lei Zheng, Joseph M. Herman, Jeffrey Meyer, Amol K. Narang

**Affiliations:** 1 Department of Radiation Oncology and Molecular Radiation Sciences, Johns Hopkins University School of Medicine, Sidney Kimmel Cancer Center, 401 N Broadway, Baltimore, MD 21231 USA; 2 Department of Radiation Oncology, Rutgers Robert Wood Johnson Medical School, 195 Little Albany Street, New Brunswick, NJ 08901 USA; 3 Philadelphia College of Osteopathic Medicine, 4170 City Ave, Philadelphia, PA 19131 USA; 4 Department of Surgery, Johns Hopkins University School of Medicine, Sidney Kimmel Cancer Center, 401 N Broadway, Baltimore, MD 21231 USA; 5 Department of Oncology, Johns Hopkins University School of Medicine, Sidney Kimmel Cancer Center, 401 N Broadway, Baltimore, MD 21231 USA; 6 Department of Radiation Oncology, Northwell Health, 450 Lakeville Road, New Hyde Park, NY 11042 USA

**Keywords:** Radiation induced lymphopenia, Vertebral body irradiation, Splenic irradiation, Stereotactic body radiation therapy, SBRT, Pancreatic cancer, Pancreatic adenocarcinoma, Vertebral body dose, Spleen dose

## Abstract

**Objectives:**

The purpose of this study was to determine if vertebral body and splenic dosimetry was associated with the development of lymphopenia in patients with borderline resectable (BRPC) and locally advanced pancreatic cancer (LAPC) treated with stereotactic body radiation therapy (SBRT).

**Methods:**

Patients with BRPC/LAPC who were treated with SBRT and who had lymphocyte counts and radiation treatment plans available for review were included in the study. Vertebral body levels T11-L3 and the spleen were retrospectively contoured for each patient. Univariate (UVA) and multivariable analyses (MVA) were performed to identify associations between vertebral body and splenic dosimetric parameters with absolute lymphocyte count (ALC) and grade ≥ 2 lymphopenia. Receiver operator characteristic curves were generated to identify dose-volume thresholds in predicting grade ≥ 2 lymphopenia.

**Results:**

A total of 132 patients were included in the study. On UVA and MVA, vertebral V15 (regression coefficient [β]: − 0.026, 95% CI − 0.044 to − 0.009, *p* = 0.003), vertebral V2.5 (β: − 0.011, 95% CI − 0.020 to − 0.002, *p* = 0.015), and log_10_PTV (β: − 0.15, 95% CI − 0.30 to − 0.005, *p* = 0.042) were associated with post-SBRT ALC. On UVA and MVA, vertebral V15 (odds ratio [OR]: 3.98, 95% CI 1.09–14.51, *p* = 0.027), vertebral V2.5 (OR: 1.04, 95% CI 1.00–1.09, *p* = 0.032), and spleen V10 (OR: 1.05, 95% CI 1.09–1.95, *p* = 0.004) were associated with development of grade ≥ 2 lymphopenia. Development of grade ≥ 2 lymphopenia was more likely in patients with vertebral V15 ≥ 5.84% (65.5% vs 34.0%, *p* = 0.002), vertebral V2.5 ≥ 48.36% (48.9% vs 23.8%, *p* = 0.005), and spleen V10 ≥ 4.17% (56.2% vs 26.9%, *p* < 0.001).

**Conclusions:**

Increasing radiation dose to vertebral bodies and spleen were associated with the development of lymphopenia in BRPC/LAPC treated with SBRT. Optimization of vertebral body and splenic dosimetry may reduce the risk of developing lymphopenia and improve clinical outcomes in this population.

**Supplementary Information:**

The online version contains supplementary material available at 10.1186/s13014-021-01969-1.

## Introduction

Pancreatic cancer is currently the third most common cause of cancer related deaths in the United States, responsible for over 48,000 deaths each year [1]. By the year 2030, it is expected to be the second most common cause of cancer related deaths [2]. Treatment of localized disease usually involves a combination of chemotherapy, radiation therapy, and/or surgical resection [3]. However, even with aggressive therapy, outcomes are poor, with 5-year overall survival (OS) rates of less than 20% for patients with non-metastatic disease [4].

Aggressive multi-modality treatment regimens can also deplete lymphocytes, which can have an impact on outcomes. Treatment related lymphopenia is seen in a wide range of malignancies including esophageal cancer, non-small cell lung cancer (NSCLC), head and neck cancer, and pancreatic cancer [5–8]. It is associated with poor survival and tumor control outcomes, likely due to the depletion of anti-tumorigenic cytotoxic T cells in both the peripheral blood and tumor microenvironment (TME) [9, 10]. The poor prognosis of pancreatic cancer can be attributed in part to its poorly immunogenic TME, which is characterized by high levels of myeloid–derived suppressor cells and low levels of cytotoxic T cells [11, 12]. This has undermined the ability to take advantage of novel therapies such as immune checkpoint inhibitors (ICIs) [13, 14]. Therefore, strategies to minimize lymphopenia should be explored to optimize outcomes in pancreatic cancer.

Radiation techniques such as intensity modulated radiation therapy (IMRT) have been used to minimize hematological toxicity by reducing dose to pelvic and lumbar spinal bone marrow in the treatment of pelvic malignancies [15, 16]. Studies have also demonstrated that sparing of thoracic spinal bone marrow can prevent lymphopenia when treating NSCLC and esophageal cancer [17, 18]. Similar findings were demonstrated when minimizing splenic dose in the treatment gastrointestinal cancers [19]. However, there have been no studies investigating the effect of vertebral body and splenic dosimetry on lymphocyte kinetics in localized pancreatic cancer treated with stereotactic body radiation therapy (SBRT). Only one report exists on the impact of unintentional splenic radiation on lymphopenia in pancreatic cancer treated with conventional chemoradiation, but other key dosimetric parameters, including size of the target volume and dose to the vertebral bodies, were not examined [20]. Therefore, the purpose of this study was to determine if vertebral body and splenic dosimetry is associated with lymphopenia in localized pancreatic cancer patients treated with SBRT, as these findings may have implications for optimizing radiation planning for pancreatic cancer.

## Methods

### Study design

This was a single-institution retrospective review of patients with localized pancreatic cancer who were treated with SBRT from August 2016 to May 2021 and who had laboratory values and radiation treatment plans available for review. Our institutional review board approved the study. The inclusion criteria for this study were as follows: (1) Biopsy proven diagnosis of pancreatic cancer, (2) Borderline resectable pancreatic cancer (BRPC) or locally advanced pancreatic cancer (LAPC) per NCCN guidelines [3], (3) Absolute lymphocyte counts (ALC) collected prior to and after SBRT, and (4) Dosimetric data and treatment plans available for review. Note that the BRPC/LAPC population was chosen given that it has been our institutional practice pattern to administer radiation therapy for patients with these stages of disease.

### Overall treatment paradigm

Patients were treated with upfront modified FOLFIRINOX (mFFX) and/or gemcitabine plus nab-paclitaxel (GnP). During chemotherapy, pancreatic protocol computed tomography (CT) scans were acquired approximately every 3 months to assess treatment response. Patients with stable or responding disease were treated with SBRT in five fractions. After completion of SBRT, patients were re-staged with imaging. All patients with BRPC were taken for surgical exploration if they did not have medical contraindications or evidence of disease progression. This was also true for LAPC patients at our institution over this time, with the exception of those with too locally advanced disease characterized by encasement of multiple vasculature structures, precluding a reasonable pathway for complete surgical resection.

### SBRT details

After completion of upfront multi-agent chemotherapy, patients were planned for SBRT. Prior to simulation, endoscopic ultrasound-guided placement of gold fiducials was performed for assistance with daily image guidance. At time of simulation, patients were positioned supine with arms above their head in a Vac-Lok (CIVCO Medical Solutions, Coralville, IA, USA) for immobilization. Thin sliced CT scans with intravenous contrast were obtained and used for treatment planning. To minimize respiratory motion, active breathing control (ABC, Elekta, Stockholm, Sweden) was utilized in the majority of patients. Patients were treated under free-breathing conditions if they could not tolerate breath-hold. These patients underwent a 4-dimensional CT scan at time of simulation, with an internal target volume (ITV) generated from the peak inspiratory and expiratory phases. Target volumes and organs at risk were delineated using Pinnacle treatment planning system (Phillips Radiation Oncology Systems, Fitchburg, WI). From 2016–2019, the clinical target volume (CTV) consisted of gross disease plus the full circumference of involved vasculature. From 2019-onward, the CTV was expanded to consist of gross disease, the full circumference of involved vasculature, and an elective volume that encompassed the celiac artery, common hepatic artery, superior mesenteric artery, portal vein, superior mesenteric vein, and the tissue between these structures. The planning target volume (PTV) was created by adding a 2–5 mm isotropic margin to the CTV in breath-hold cases or to the ITV in free-breathing cases. Planning objectives were as follows: (1) dose coverage—prescription dose to cover at least 98% of CTV and 90% of PTV, 25 Gy to cover 100% of CTV and at least 99% of PTV, (2) gastrointestinal structures (stomach, duodenum, small/large bowel)—V33 < 1 cc, V20 < 20 cc, max dose (Dmax) < 40 Gy, (3) combined kidneys –V12 < 25%, (4) liver—V12 < 50% and greater than 700 cc receiving less than 15 Gy, (5) spinal canal—V8 < 1 cc. Radiation dose was prescribed to the 70–90% isodose line. Pre-treatment and intrafraction cone-beam CT scans were performed to confirm and monitor appropriate patient setup. Patients were aligned to spine and then shifted to align to fiducials. All treatments were performed on an Elekta linear accelerator unit (Elekta, Stockholm, Sweden). Approximately four weeks after completion of SBRT, patients underwent re-staging imaging followed by surgical exploration if deemed appropriate by the surgical team. The initiation of adjuvant or maintenance chemotherapy was at the discretion of the treating medical oncologist.

### Laboratory values

Complete blood cell count with differential were reviewed for each patient. Absolute lymphocyte counts were recorded within 4 weeks prior to SBRT and 1–6 weeks after completion of SBRT. If multiple values existed, the value closest to the start of SBRT and closest to 4 weeks after completion of SBRT were recorded. Lymphopenia was graded according to the common terminology of adverse events (CTCAE) [21]: grade 1 (1.0–0.8 no./µL), grade 2 (< 0.8–0.5 no./µL), grade 3 (< 0.5–0.2 no./µL), and grade 4 (< 0.2 no./µL).

### Radiation volumes and dosimetry

Vertebral bodies and the spleen were retrospectively contoured for each patient using Pinnacle treatment planning system (Phillips Radiation Oncology Systems, Fitchburg, WI). All plans were reviewed prior to contouring to identify vertebral body levels that were at least one level above and below the superior and inferior aspects of the PTV, respectively, so that meaningful dose to the vertebral bodies could be captured. It was determined that vertebral body levels T11-L3 included this region and was subsequently contoured on the planning the CT scan for each patient. Dosimetric data for vertebral V2.5-V20 and splenic V2.5-V15 in 2.5 Gy increments were collected from dose volume histograms and Pinnacle treatment planning scorecards. Mean vertebral body and splenic dose were also recorded. Planning target volume was included in the analysis to account for potential dose to lymph nodes/lymphatic channels and circulating lymphocytes through the radiation field.

### Statistical analysis

Descriptive statistics were used to record patient, treatment, and disease characteristics such as age, sex, Eastern Cooperative Oncology Group performance status, histology, tumor location, disease extent, chemotherapy regimen, SBRT regimen, surgical status, laboratory values, and dosimetric parameters. Absolute lymphocyte count and PTV values were log transformed for normalization of data. Univariate and multivariable linear regression were performed to identify variables associated with log-transformed post-SBRT lymphocyte counts. Similarly, univariate and multivariable logistic regression were performed to identify variables associated with development of grade ≥ 2 lymphopenia. Variables with *p* < 0.2 on univariate analyses were entered into multivariable analyses and subsequently removed in a stepwise manner if P value became > 0.2. Because this analysis included numerous closely related dosimetric parameters, collinearity was present. Variables were excluded if they displayed a high degree of collinearity (i.e. if their regression coefficient (β) flipped signs or odds ratio became inverted when included in multivariable analyses). Receiver operating characteristic curves were generated to identify the optimal dose-volume thresholds in predicting grade ≥ 2 lymphopenia. Threshold values were selected based on the maximum Youden index (sensitivity + specificity-1). A *P* value < 0.05 was considered statistically significant during the study, and all P values were two-sided. Statistical analyses were performed with JMP version 15.0 (SAS institute, Cary NC, USA) and SPSS version 25.0 (IBM Corporation, Armonk NY, USA).

## Results

### Patient characteristics

A total of 132 patients were treated with SBRT for localized pancreatic cancer and had both laboratory values and dosimetric information available for review. Table 1 displays patient characteristics of the cohort. The median age was 65.5 years (range 41.7–84.1 years). Adenocarcinoma was the predominant histology (131/132, 99.2%), with one patient having undifferentiated carcinoma. Borderline resectable disease was found in 41 patients (31.1%) and locally advanced disease in 91 patients (69.9%). All patients received induction chemotherapy with either mFFX (103/132, 78%), GnP (21/132, 15.8%), mFFX and GnP (5/132, 3.8%), or other regimens (3/132, 2.4%). Nearly all patients received SBRT to 33 Gy in 5 fractions (128/132, 97.0%), with other regimens including 30 Gy in 5 fractions (2/132, 1.5%), 36 Gy in 5 fractions (1/132, 0.8%), and 30.5 in 5 fractions (1/132, 0.8%). Surgical resection was performed in 90 patients (68.2%), which included the Whipple procedure (54/90, 60.0%), distal pancreatectomy (31/90, 34.4%), or total pancreatectomy (5/90, 5.6%).

**Table 1:** Patient, treatment, and disease characteristics

Characteristics	N (%) or median (range)
No. of patients	132
Age (years)	65.5 (41.7–84.1)
*Sex*	
Male	75 (56.8)
Female	57 (43.2)
*ECOG*	
0	50 (37.9)
1–2	82 (62.1)
*Histology*	
Adenocarcinoma	131 (99.2)
Undifferentiated carcinoma	1 (0.8)
*Location of primary tumor*	
Head	59 (44.7)
Other	73 (55.3)
*Disease extent*	
Borderline resectable	41 (31.1)
Locally advanced	91 (69.9)
Baseline CA 19-9 (U/mL)	183.9 (1.0–7358.4)
Induction chemotherapy duration (months)	4 (1–18)
*Induction chemotherapy regimen*	
mFFX	103 (78.0)
GnP	21 (15.8)
mFFX and GnP	5 (3.8)
mFFX plus other	1 (0.8)
GnP plus other	1 (0.8)
Other	1 (0.8)
*SBRT dose and fractionation*	
33 Gy in 5 fractions	128 (97.0)
30 Gy 5 fractions	2 (1.4)
36 Gy in 5 fractions	1 (0.8)
30.5 Gy in 5 fractions	1 (0.8)
PTV (cm^3^)	132.0 (13.1–428.3)
Surgically Resected	90 (68.2)
Whipple procedure	54 (60.0)
Distal prancreatectomy	31 (34.4)
Total pancreatectomy	5 (5.6)

*ECOG* Eastern Cooperative Oncology Group, *CA 19-9*, carbohydrate antigen 19-9, *mFFX* modified FOLFIRINOX, *GnP* gemcitabine/nab-paclitaxel, *SBRT* stereotactic body radiation therapy, *PTV* planning target volume, *ALC* absolute lymphocyte count

### Lymphocyte counts and dosimetric parameters

Table 2 displays information on lymphocyte counts and dosimetric parameters. Median pre-SBRT ALC was 1.46 no./uL (range 0.33–3.73 no./uL), and median post-SBRT was 0.83 no./uL (range 0.18–1.81 no./uL), resulting in a change of − 43.2% (Mann–Whitney U, *p* < 0.001). Overall, 54 patients (40.9%) developed grade ≥ 2 lymphopenia. Median values for vertebral V20, V17.5, V15, V12.5, V10, V7.5, V5, and V2.5 were 0% (range 0–14.83%), 0.16% (range 0–24.74%), 1.69% (range 0–36.19%), 6.38% (range 0–44.68%), 17.03% (range 0.07–51.14%), 31.54% (range 7.45–67.73%), 43.03% (range 15.02–81.18%), and 53.84% (range 26.14–91.08%), respectively. The median mean vertebral dose was 5.12 Gy (range 2.23–11.14 Gy). Median values for spleen V15, V12.5, V10, V7.5, V5, and V2.5 were 0% (range 0–29.16%), 0.15% (range 0–40.69%), 3.71% (range 0–61.63%), 18.14% (range 0–83.06%), 42.58% (range 0–92.15%), and 61.04% (range 0–99.96%), respectively. The median mean spleen dose was 4.46 Gy (range 0.30–11.72 Gy).

**Table 2:** Summary of lymphocyte data and dosimetric parameters

Variable	N (%) or median (range)
*Absolute lymphocyte counts (no./µL)*	
Pre-SBRT	1.46 (0.33–3.73)
Post-SBRT	0.83 (0.18–1.81)
*Grade* *≥* *2 lymphopenia*	
Yes	54 (40.9)
No	78 (59.1)
VertebralV20 (%)	0 (0.0–14.83)
VertebralV17.5 (%)	0.16 (0.0–24.74)
VertebralV15 (%)	1.69 (0.0–36.19)
VertebralV12.5 (%)	6.38 (0.0–44.68)
VertebralV10 (%)	17.03 (0.07–51.14)
VertebralV7.5 (%)	31.54 (7.45–67.73)
VertebralV5 (%)	43.03 (15.02–81.18)
VertebralV2.5 (%)	53.84 (26.14–91.08)
Mean vertebral dose (Gy)	5.12 (2.23–11.14)
SpleenV15 (%)	0 (0.0–29.16)
SpleenV12.5 (%)	0.15 (0.0–40.69)
SpleenV10 (%)	3.71 (0.0–61.63)
SpleenV7.5 (%)	18.14 (0.0–83.06)
SpleenV5 (%)	42.58 (0.0–92.15)
SpleenV2.5 (%)	61.04 (0.0–99.96)
Mean spleen dose (Gy)	4.46 (0.30–11.72)

### Predictors of post-SBRT absolute lymphocyte count

Table 3 shows univariate and multivariable linear regression of log-transformed post-SBRT ALC. On univariate analysis, log_10_PTV, vertebral dosimetric parameters (V2.5–20 and mean), and splenic dosimetric parameters (V2.5–15 and mean) were all negatively associated with log-transformed post-SBRT ALC. On MVA, only log_10_PTV (β: − 0.15, 95% CI − 0.30 to − 0.005, *p* = 0.042), vertebral V15 (β: − 0.026, 95% CI − 0.044 to − 0.009, *p* = 0.003), and vertebral V2.5 (β: − 0.011, 95% CI − 0.020 to − 0.002, *p* = 0.015) were associated with log-transformed post-SBRT ALC. To assess whether these associations were present prior to radiation or were radiation induced, log_10_PTV, vertebral V15, and vertebral V2.5 were plotted against log-transformed pre-SBRT lymphocyte counts, with no correlation detected (Additional file 1: Figure 1).

**Table 3:** Univariate and multivariable linear regression of log-transformed post-SBRT absolute lymphocyte count

	UVA			MVA		
	β	95% CI	*P*	β	95% CI	*P*
Age (years)	− 0.001	− 0.004 to 0.002	0.535			
Sex (male vs female)	− 0.012	− 0.042 to 0.019	0.440			
ECOG (0 vs 1–2)	− 0.006	− 0.038 to 0.025	0.683			
Disease extent (BRPC vs LAPC)	− 0.002	− 0.035 to 0.030	0.892			
Induction CT duration (months)	− 0.006	− 0.020 to 0.007	0.357			
Log_10_PTV	− 0.13	− 0.216 to − 0.037	0.006	− 0.15	− 0.30 to − 0.005	0.042
VertebralV20 (%)	− 0.013	− 0.025 to − 0.001	0.032			
VertebralV17.5 (%)	− 0.009	− 0.017 to 0.001	0.022			
VertebralV15 (%)	− 0.006	− 0.011 to − 0.001	0.013	− 0.026	− 0.044 to − 0.009	0.003
VertebralV12.5 (%)	− 0.004	− 0.008 to − 0.001	0.019			
VertebralV10 (%)	− 0.003	− 0.005 to − 2e−5	0.049			
VertebralV7.5 (%)	− 0.002	− 0.004 to 4e−5	0.054			
VertebralV5 (%)	− 0.003	− 0.005 to − 3e−4	0.027			
VertebralV2.5 (%)	− 0.003	− 0.005 to − 2e−4	0.031	− 0.011	− 0.020 to − 0.002	0.015
Mean vertebral dose (Gy)	− 0.001	− 4e−4 to − 5e−5	0.014			
SpleenV15 (%)	− 0.005	− 0.011 to 0.001	0.090			
SpleenV12.5 (%)	0.002	− 0.007 to 1e−4	0.061			
SpleenV10 (%)	− 0.002	− 0.005 to − 3e−4	0.027	− 0.006	− 0.013 to 8e−4	0.084
SpleenV7.5 (%)	− 0.002	− 0.003 to − 2e−4	0.028			
SpleenV5 (%)	− 0.002	− 0.003 to − 2e−4	0.019	− 0.005	− 0.011 to 5e−4	0.072
SpleenV2.5 (%)	− 0.001	− 0.002 to 4e−5	0.058			
Mean spleen dose (Gy)	1e−4	− 3e−4 to − 2e−5	0.021			

*ECOG* Eastern Cooperative Oncology Group, *BRPC* borderline resectable pancreatic cancer, *LAPC* locally advanced pancreatic cancer, *CT* chemotherapy, *PTV* planning target volume, *SBRT* stereotactic body radiation therapy, *ALC* absolute lymphocyte count

### *Predictors of grade* ≥ *2 lymphopenia*

Given that that log_10_PTV, vertebral V15, and vertebral V2.5 were negatively associated with post-radiation lymphocyte count, we next wanted to determine if these variables also predicted for grade ≥ 2 lymphopenia. Table 4 shows univariate and multivariable logistic regression of grade ≥ 2 lymphopenia. On univariate logistic regression, log_10_PTV, vertebral dosimetric parameters (V2.5–20 and mean), and splenic dosimetric parameters (V2.5–15 and mean) were significantly associated with the development of grade ≥ 2 lymphopenia. However, on multivariable logistic regression, only vertebral V15 (odds ratio [OR]: 3.98, 95% CI 1.09–14.51, *p* = 0.027), vertebral V2.5 (OR: 1.04, 95% CI 1.003–1.09, *p* = 0.032), and spleen V10 (OR: 1.05, 95% CI 1.09–1.95, *p* = 0.004) were associated with development of grade ≥ 2 lymphopenia.

**Table 4:** Univariate and multivariable analysis for predictors of grade ≥ 2 lymphopenia

	UVA			MVA		
	OR	95% CI	*P*	OR	95% CI	*P*
Age (years)	1.03	0.99–1.07	0.182			
Sex (male vs female)	0.48	0.24–0.98	0.044			
ECOG (0 vs 1–2)	1.23	0.60–2.51	0.573			
Disease extent (BRPC vs LAPC)	0.57	0.26–1.23	0.151			
Induction CT duration (months)	1.04	0.89–1.22	0.648			
Log_10_PTV	5.34	1.60–17.81	0.004			
VertebralV20 (%)	1.19	1.42–0.84	0.027	4.02	0.55–29.41	0.158
VertebralV17.5 (%)	1.13	1.01–1.27	0.017			
VertebralV15 (%)	1.09	1.02–1.17	0.009	3.98	1.09–14.51	0.027
VertebralV12.5 (%)	1.06	1.01–1.10	0.011			
VertebralV10 (%)	1.04	1.00–1.07	0.024			
VertebralV7.5 (%)	1.03	1.00–1.06	0.033			
VertebralV5 (%)	1.03	1.00–1.06	0.027			
VertebralV2.5 (%)	1.03	1.00–1.06	0.023	1.04	1.00–1.09	0.032
Mean vertebral dose (Gy)	1.00	1.00–1.00	0.007			
SpleenV15 (%)	1.11	1.01–1.22	0.010			
SpleenV12.5 (%)	1.07	1.01–1.12	0.005			
SpleenV10 (%)	1.05	1.02–1.08	0.001	1.05	1.09–1.95	0.004
SpleenV7.5 (%)	1.03	1.01–1.05	0.001			
SpleenV5 (%)	1.03	1.01–1.04	0.001			
SpleenV2.5 (%)	1.02	1.01–1.04	0.004			
Mean spleen dose (Gy)	1.00	1.00–1.00	0.001			

*ECOG* Eastern Cooperative Oncology Group, *BRPC* borderline resectable pancreatic cancer, *LAPC* locally advanced pancreatic cancer, *CT* chemotherapy, *PTV* planning target volume, *SBRT* stereotactic body radiation therapy, *ALC* absolute lymphocyte count

### *Dosimetric thresholds of predicting grade* ≥ *2 lymphopenia*

**Fig. 1:**
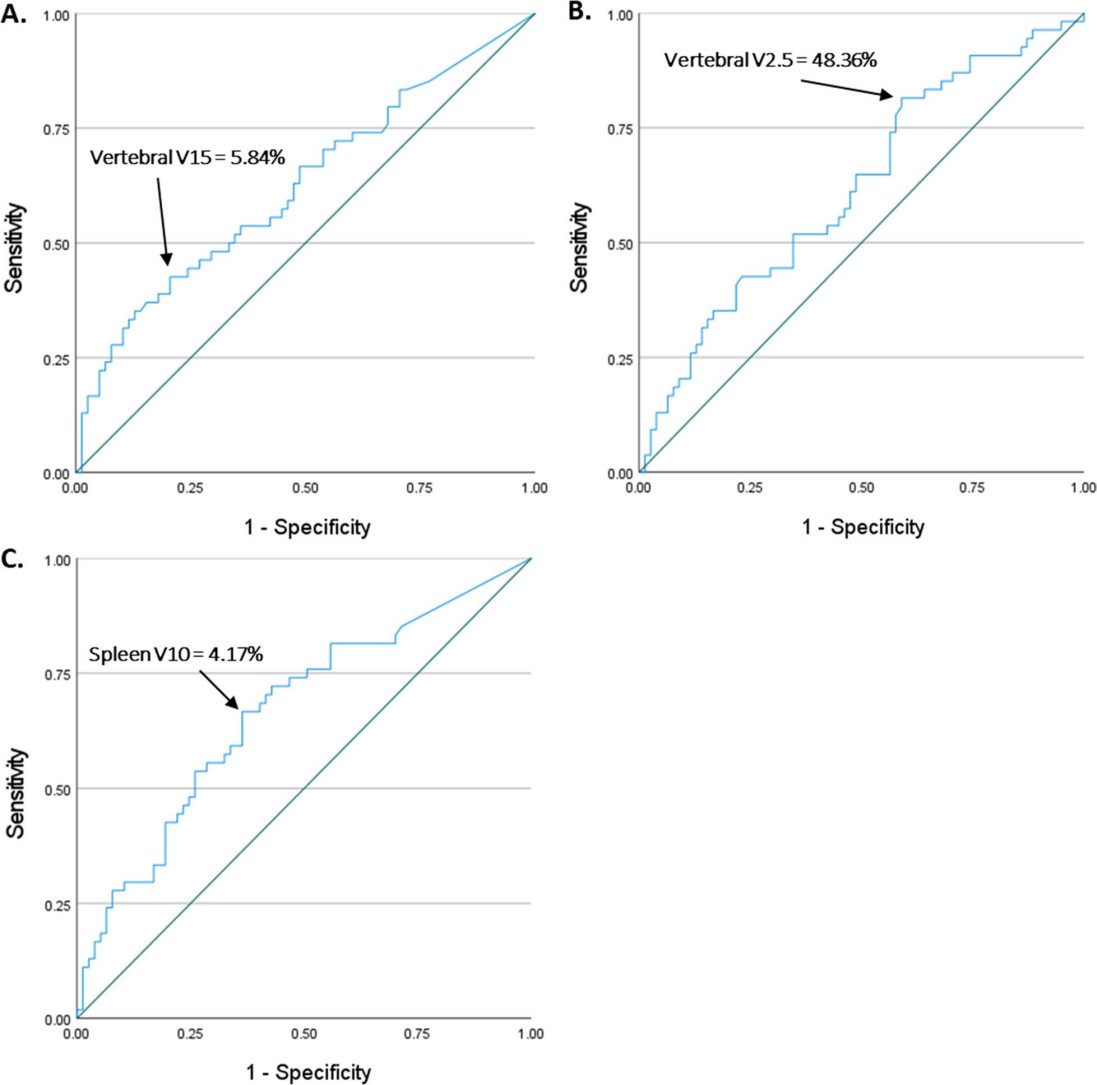
Receiver operating characteristic curves showing optimal thresholds for vertebral V15 (**a**), vertebral V2.5 (**b**), and spleen V10 (**c**) in predicting development of grade ≥ 2 lymphopenia

On multivariable analyses, both vertebral V15 and vertebral V2.5 were significantly associated with post-SBRT ALC (continuous variable) and development of grade ≥ 2 lymphopenia (categorical variable), while spleen V10 was associated with development of grade ≥ 2 lymphopenia. Therefore, we wanted to identify thresholds for these three dosimetric parameters in predicting grade ≥ 2 lymphopenia, which may guide clinicians during the radiation planning process. Figure 1a–c show receiver operating characteristic curves with the optimal cutoff values in predicting grade ≥ 2 lymphopenia. The optimal thresholds for vertebral V15, vertebral V2.5, and spleen V10 in predicting grade ≥ 2 lymphopenia were 5.84% (area under curve [AUC]: 0.62 sensitivity: 35.2% specificity: 87.2%), 48.36% (AUC: 0.62 sensitivity: 81.5% specificity: 59.0%), and 4.17% (AUC: 0.67, sensitivity: 66.7%, specificity: 63.6%), respectively. Development of grade ≥ 2 lymphopenia was more likely in patients with vertebral V15 ≥ 5.84% (65.5% vs 34.0%, *p* = 0.002), vertebral V2.5 ≥ 48.36% (48.9% vs 23.8%, *p* = 0.005), and spleen V10 ≥ 4.17% (56.2% vs 26.9%, *p* < 0.001) (Table 5).

**Table 5:** Development of grade ≥ 2 lymphopenia based on vertebral V15, vertebral V2.5, and spleen V10 thresholds

Vertebral V15	Grade ≥ 2 lymphopenia		*P* value
	Yes, n (%)	No, n (%)	
< 5.84%	35 (34.0%)	68 (66.0%)	0.002
≥ 5.84%	19 (65.5%)	10 (34.5%)	

Vertebral V2.5	Grade ≥ 2 lymphopenia		*P* value
	Yes, n (%)	No, n (%)	
< 48.36%	10 (23.8%)	32 (76.2%)	0.005
≥ 48.36%	44 (48.9%)	46 (51.1%)	

Spleen V10	Grade ≥ 2 lymphopenia		*P* value
	Yes, n (%)	No, n (%)	
< 4.17%	18 (26.9%)	49 (73.1%)	< 0.001
≥ 4.17%	36 (56.2%)	28 (43.8%)	

**Fig. 2:**
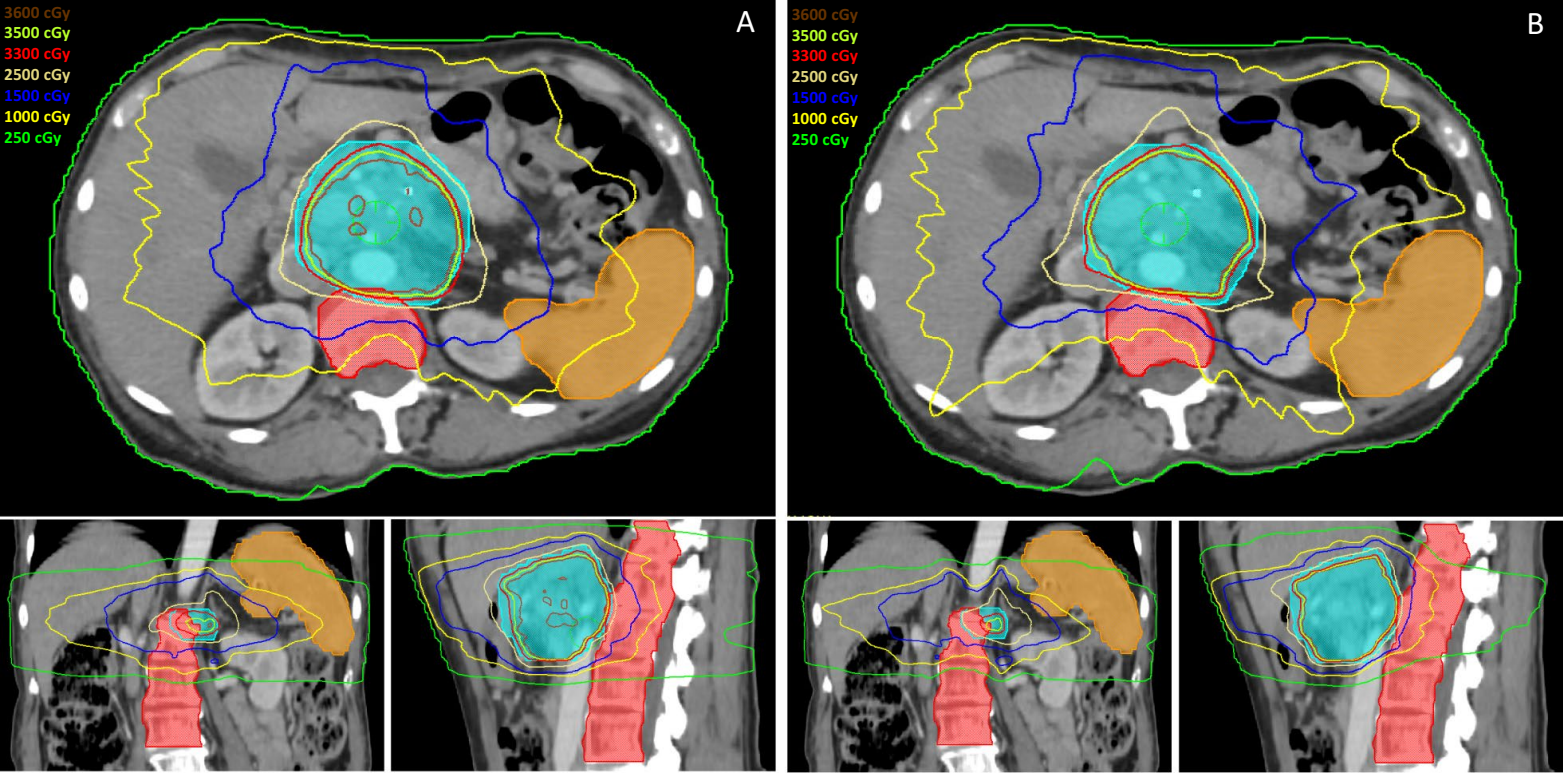
Radiation treatment plan with isodose lines on axial, coronal, and sagittal planning images for **a** initial plan which exceeded vertebral body and splenic thresholds and **b** optimized plan which met vertebral body and splenic constraints. Turquoise colorwash represents PTV, orange colorwash represents spleen, and red colorwash represents vertebral bodies. Isocenter represented by green crosshair on axial image

**Fig. 3:**
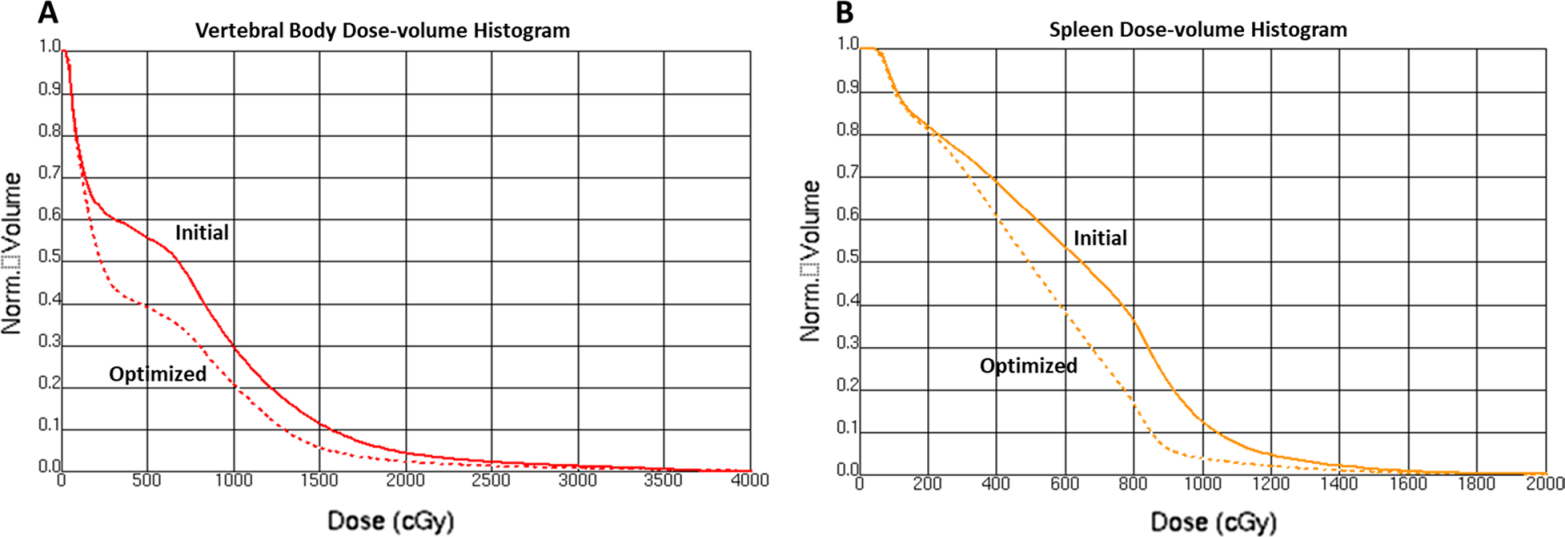
Dose-volume histograms for **a** vertebral body and **b** spleen for the initial (solid line) and optimized (dashed line) plans

We next wanted to explore if the above thresholds could have been achieved in patients who were treated clinically with plans that did not meet these thresholds, while still achieving all other planning objectives. As such, we re-planned a patient whose initial radiation plan exceeded the aforementioned vertebral body and splenic thresholds (vertebral V15 = 11.30%, V2.5 = 61.57%, spleen V10 = 12.30%). After optimization, vertebral body and splenic constraints were successfully achieved (vertebral V15 = 5.55%, vertebral V2.5 = 47.39%, spleen V10 = 3.70%) while still meeting all initial planning objectives. Figure 2a, b shows the initial and optimized plans, while Fig. 3a, b shows the dose-volume histogram for both plans.

## Discussion

In this study, we show that in a cohort of patients with BRPC or LAPC who underwent SBRT after upfront chemotherapy and for whom vertebral body and splenic dose were not part of the optimization parameters, considerable variation existed with respect to vertebral body and splenic dose. Moreover, increasing radiation dose to vertebral bodies and spleen was associated with decreased lymphocyte count and the development of grade ≥ 2 lymphopenia. More specifically, low-dose vertebral body parameters, specifically the vertebral V15 and vertebral V2.5, were associated with lymphocyte count and development of grade ≥ 2 lymphopenia, while low-dose splenic parameters, specifically the spleen V10, was associated with the development of grade ≥ 2 lymphopenia. As such, vertebral body and splenic dose may have a role in radiation planning for pancreatic cancer, and continued work should be pursued to understand optimal dose goals for these structures.

Hematopoiesis predominantly occurs in bone marrow followed by in lymphoid tissue such as the spleen, lymph nodes, and thymus. Radiation induced lymphopenia is thought to result from unintentional dose to hematopoietic organs as well as circulating lymphocytes in the blood stream and lymphatic channels. The extreme radiosensitivity of lymphocytes contributes to radiation induced lymphopenia, with single doses of 2 Gy and 3 Gy being shown to kill up to 50% and 90% of lymphocytes in vitro, respectively [22]. Given data highlighting the importance of lymphocytes in tumor control, radiation techniques to spare bone marrow such as IMRT and SBRT are now commonly utilized [15, 16, 23].

Lymphopenia has been associated with inferior survival and tumor control outcomes in a variety of malignancies including glioblastoma, NSCLC, head and neck cancer, esophageal cancer, and pancreatic cancer [9]. A study by Wild et al. demonstrated that development of grade ≥ 3 lymphopenia was associated with worse OS in LAPC treated with chemoradiation [10]. The neutrophil-to-lymphocyte ratio (NLR), which is highly dependent on lymphocyte count, is also a predictor of outcomes in pancreatic cancer [24–28]. A recent study of localized pancreatic cancer treated with radiation showed that an increase in NLR was primarily due to depletion of lymphocytes and associated with decreased OS and surgical resection rates [25]. Data from our institution (not yet published) corroborate these findings. The exact mechanism of how lymphocytes improve tumor control is not known, but it is thought to be due direct effects of cytotoxic T lymphocytes on cancer cells [29].

Dosimetric objectives that can be used during the radiation planning process to minimize lymphopenia would be useful in the treatment of pancreatic cancer, which is characterized by poor tumor immunogenicity. Here we show that vertebral V15, vertebral V2.5, and spleen V10 are all predictive of developing grade ≥ 2 lymphopenia and may have utility in dosimetric planning. Vertebral body dosimetry has been shown to be associated with development of lymphopenia in both NSCLC and esophageal cancer [17, 18]. For example, Deek et al. showed the utility of thoracic vertebral V20, V30 and mean dose in NSCLC [18]. However, our report is the first to show an association between vertebral dosimetry and lymphopenia in pancreatic cancer treated with SBRT. Our findings on splenic dosimetry are consistent with a report by Chadha et al., who demonstrated that mean spleen dose and spleen V15 were associated with ≥ grade 3 lymphopenia in LAPC treated with chemoradiation [20]. In our study, very few patients developed ≥ grade 3 lymphopenia (11/132, 8.3%), potentially due to higher conformality with SBRT, as compared to IMRT and three-dimensional conformal radiation (3D-CRT) [23, 30]. Moreover, while the specific dose thresholds that were significant in our cohort differed numerically compared to the aforementioned findings in the NSCLC and pancreatic cancer settings, part of this may be explained by the fact that patients in our cohort were treated in a hypo-fractionated manner. Nonetheless, our findings suggest that vertebral body and splenic dosimetry should be optimized to reduce the risk of lymphopenia in pancreatic cancer patients treated with SBRT. Future studies should continue to examine optimal dose thresholds for these structures. Additionally, optimal thresholds in the setting of dose-escalated radiation should also be defined [31].

We also demonstrate that larger PTVs are associated with decreased post-SBRT lymphocyte counts, consistent with findings from other studies [10, 32, 33]. This may have implications on radiation field design. Currently, there is no consensus on optimal radiation volumes in the treatment of intact pancreatic cancer. Some advocate for the treatment of gross disease plus involved vasculature while others suggest that there may be a benefit in treating a larger volume that includes gross disease, involved vasculature, and elective nodal regions [34–37]. Our findings show that treating to larger volumes may deplete lymphocyte counts, which in turn, may negatively impact clinical outcomes. Of note, although PTV was associated with lymphocyte count, it was not associated with the development of grade ≥ 2 lymphopenia, suggesting that it may not be as clinically relevant as vertebral body and splenic dose, which did predict for grade ≥ 2 lymphopenia. Given these findings, one potential approach may involve treating to larger volumes but optimizing vertebral body and splenic dosimetry to offset potential impact on lymphopenia of a larger target volume. Ultimately, further investigation is needed to determine how radiation field design impacts lymphocyte counts and how this may translate to clinical outcomes.

Our findings may also have relevance to pancreatic cancer patients who are treated with SBRT and immunotherapy. Although immunotherapy has shown promise in a wide range of malignancies, monotherapy with ICIs has shown to have little benefit [13, 14]. This is likely due to the immunosuppressive and hypoxic environment of the pancreatic TME. To increase tumor immunogenicity, a number of current trials are investigating combination therapy of ICIs with SBRT, chemokine inhibitors, oncolytic viruses, and vaccines [38, 39]. Many of these novel agents act to kill cancer cells through direct activation of lymphocytes. Therefore, preservation of lymphocytes in this setting may be especially important. Our data suggests that limiting vertebral body and splenic dose may prevent clinically significant lymphopenia. As a result, patients being treated on combination therapy trials may derive the greatest benefit from optimization of vertebral body and splenic dosimetry. This scenario may also prove to be a setting in which elective regions are omitted from the target volume. Certainly, more data is needed to help inform such decision-making.

This study has several limitations including its retrospective design. Patients received various neoadjuvant chemotherapy regimens, which in turn, may have influenced laboratory values. Lymphocyte counts were also recorded anywhere from 1 to 6 weeks following SBRT. It is possible that these values may have fluctuated during this interval. Furthermore, time interval between chemotherapy and SBRT as well as the development of lymphopenia during chemotherapy would have both impacted post-SBRT lymphocyte counts. Unfortunately, this information was not available for review since many patients received chemotherapy at outside institutions. In addition, because multiple closely related dosimetric parameters were analyzed, there was some degree of collinearity, which likely influenced statistical significance of certain variables. Moreover, future studies should examine the implications constraining vertebral body and splenic dose with respect to dose to other organs at risk as well as magnitude of hotspots. The strengths of this study include its large study population (n = 132) and homogenous SBRT regimen of 33 Gy in 5 fractions (128/132, 97%). Despite the study’s limitations, these findings are consistent with reports from others and adds novel information on this topic.

To our knowledge, this is the first study to investigate the impact of vertebral body and splenic dosimetry on lymphopenia in localized pancreatic cancer treated with SBRT. Increasing radiation dose to vertebral bodies and the spleen were associated with lymphopenia. More specifically, vertebral V15 and vertebral V2.5 were associated with lymphocyte count and development of grade ≥ 2 lymphopenia, while spleen V10 was associated with development of grade ≥ 2 lymphopenia. These findings may have implications in the radiation planning process to reduce the risk of lymphopenia.

## Supplementary Information

**Additional file 1: Figure 1**. Correlations among log10PTV (A), vertebral V15 (B), and vertebral V2.5 (C) with log-transformed pre-SBRT ALC.

## Data Availability

The datasets generated and/or analysed during the current study are not publicly available because our IRB has not approved of sharing our patient data outside of our institution. If data is requested, we can share after approval from our IRB.
