# Supplementary figures and images for: Vertebral body and splenic irradiation are associated with lymphopenia in localized pancreatic cancer treated with stereotactic body radiation therapy
Source: Radiat Oncol. 2021 Dec 24;16:242. doi: 10.1186/s13014-021-01969-1 (PMC8709967; doi:10.1186/s13014-021-01969-1)

## Slide 1
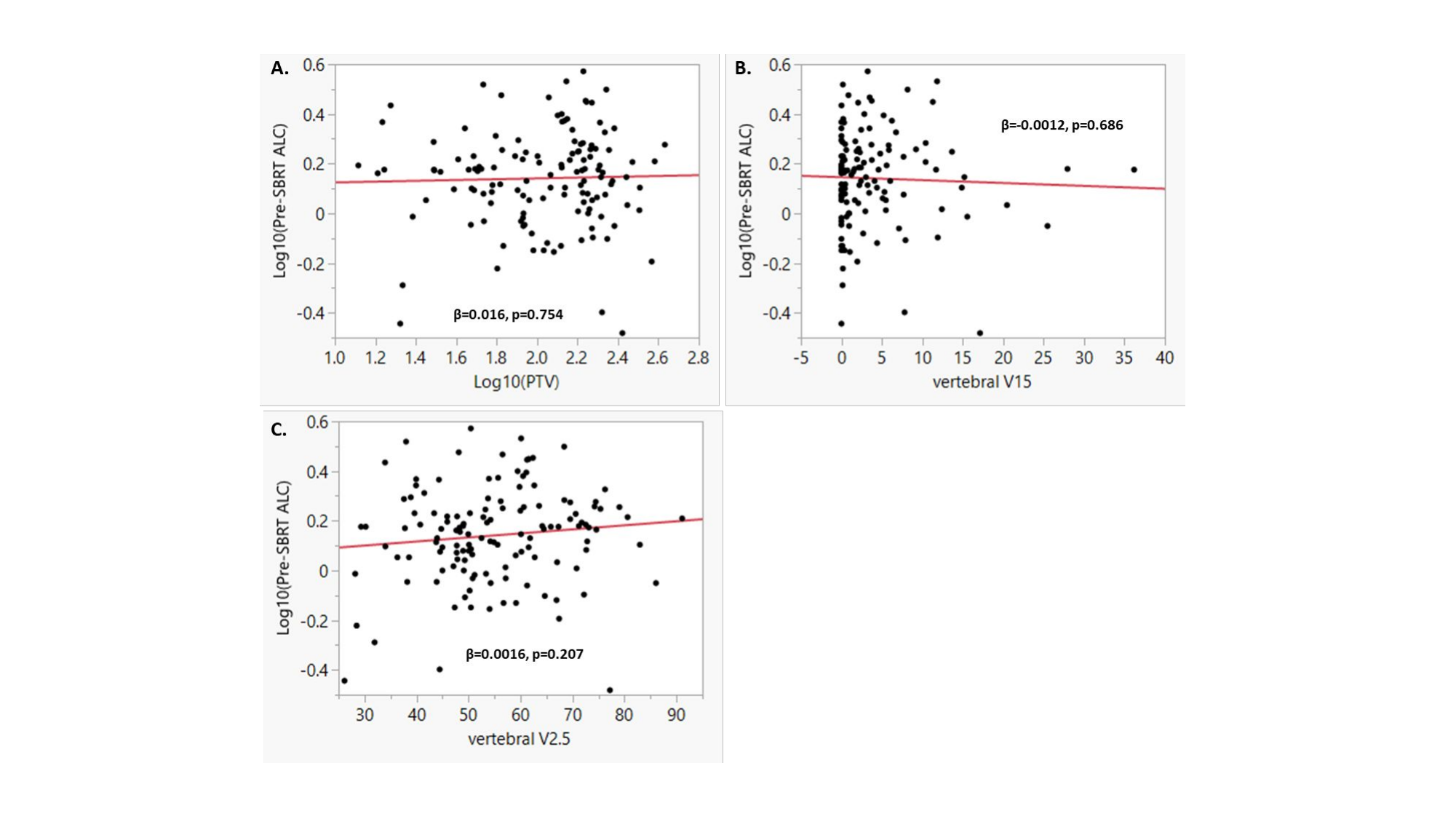

Supplement: Supplementary file 1 — Additional file 1: Figure 1. Correlations among log10PTV (A), vertebral V15 (B), and vertebral V2.5 (C) with log-transformed pre-SBRT ALC. [file 13014_2021_1969_MOESM1_ESM.pptx]
